# Revise the cognitive model using structural equation modeling

**DOI:** 10.3389/fpsyg.2026.1758968

**Published:** 2026-05-18

**Authors:** Shiwei Lin, Xiaolong Zhang, Guoxu Chen, Lingchen Sun, Xinyu Wang, Yongshuang Ma

**Affiliations:** 1 College of Chemistry, Changchun Normal University, Changchun, Jilin, China; 2 Discipline Construction Office, Changchun Normal University, Changchun, Jilin, China; 3 School of Psychology, Inner Mongolia Normal University, Hohhot, Inner Mongolia, China; 4 College of Science Education, Changchun Normal University, Changchun, Jilin, China

**Keywords:** cognitive diagnosis (CD), cognitive model (CM), middle school chemistry, revision, structural equation modeling (SEM)

## Abstract

**Background:**

Qualitative revision of the Cognitive Model (CM) is prone to subjective bias, while classical quantitative methods can only indicate model-data misfit without offering specific revision guidance. Therefore, inadequate CM fit remains a common challenge in Cognitive Diagnosis (CD) research.

**Purpose:**

This study aims to determine the CM while avoiding both the loss of examination of disciplinary connotation due to pure data-driven approaches and the potential biases that may arise from pure qualitative research.

**Method:**

Using Structural Equation Modeling (SEM) as platform, this study analyzed the valid response data from 752 students by leveraging information such as *r* and Modification Index (MI). Additionally, it incorporated the judgments of experts regarding the disciplinary connotations. Through these efforts, the preliminary CM for the content of “Composition and Structure of Substances” in middle school chemistry was revised.

**Result:**

The revised CM achieved improved fit and aligned with disciplinary connotations.

**Conclusion:**

This study employs a mixed-method SEM approach. It avoids two pitfalls. Pure data-driven methods tend to deviate from disciplinary connotations, and pure qualitative analyses are prone to subjective judgment. The resulting benefits are better data-model fit, ensured disciplinary connotations of the CM, and operational efficiency. Meanwhile, it also has drawbacks related to measurement error and sample generalizability.

## 1 Introduction

Cognitive Diagnosis (CD) shifts the focus of assessment from broad ability levels to fine-grained cognitive structures. Central to CD is the specification of a Cognitive Model (CM), a hypothesized network of cognitive attributes (knowledge, skills, and strategies) and their hierarchical relationships (Leighton et al., 2004). Ideally, the CM should reflect the actual psychological and logical sequences in problem-solving (Vosniadou and Brewer, 1992). However, in practice, the CM is typically specified by experts based on their domain expertise and teaching experience, a process that is inherently subjective and prone to variation. Consequently, for the same content domain, multiple plausible CMs may co-exist (Cheng, 2009), raising a fundamental validity question: which CM is superior? Existing empirical methods for evaluating CMs, such as the Hierarchy Consistency Index (HCI), primarily assess the degree of fit between a hypothesized CM and observed response data. While useful for model selection, these fit indices offer limited diagnostic information about why a CM misfits or how to revise its hierarchical structure. The critical gap, therefore, is the absence of an empirically guided, data-driven approach for refining the CM determined by experts, beyond simply accepting or rejecting a priori expert hypotheses.

Structural Equation Modeling (SEM) utilizes covariance structures to conduct factor analysis and path analysis (Loehlin and Beaujean, 2016; Quintana and Maxwell, 1999), thereby enabling the evaluation of consistency between theoretical hypotheses and empirical data (Bollen and Lennox, 1991; Bollen and Long, 1993; Maxwell and Delaney, 2004). It has been widely used to test theoretical models in management, psychology, and education (e.g., Leong et al., 2020; Pongsophon, 2025; Frawley and Campbell, 2025). Given that the CM postulates specific interrelationships among cognitive attributes, SEM would seem well-suited for empirically evaluating the plausibility of these hierarchical structures. However, to the best of our knowledge, no recent study has systematically reported on the method of revising the CM using SEM. Our previous work used SEM to revise the Q-matrix (Lin et al., 2025), i.e., to determine which attributes each item measures. The present study addresses a conceptually distinct and theoretically prior question: how are the cognitive attributes themselves interrelated? This question concerns the CM, which provides the foundational hierarchy upon which the Q-matrix is built. Even a perfectly specified Q-matrix cannot yield valid diagnostic inferences if the underlying CM is misspecified. Therefore, refining the CM represents a deeper level of model development, one that directly addresses the structural validity of CD research.

## 2 Method

### 2.1 Instruments

A cognitive diagnostic assessment tool for “Composition and Structure of Matter” (one of the five themes in the junior high school chemistry curriculum in China) was developed. The items in the tool are compiled based on the national curriculum standards, textbook analysis, and consultations. Subsequently, they are reviewed by an expert panel composed of three experienced junior high school chemistry teachers. All disagreements were resolved through discussion. Dichotomous scoring was used (1 = fully correct, 0 = omission or incorrect). The initial 35-item assessment was piloted on 205 students. Problematic items (with low discrimination or poor fit) were eliminated, resulting in a final 31-item version. The final assessment showed high internal consistency (Cronbach's α = 0.899). Two sources of evidence supported construct validity. Each student's predicted total score was computed using the GDINA model. The Pearson correlation between predicted and observed total scores was 0.816, indicating strong predictive validity. Second, the HCI was 0.721, confirming that the response data were consistent with the hypothesized attribute hierarchy. Extreme group analysis showed significant differences between the bottom and top 27% on all items (*t* > 3, *p* < 0.001) (Thissen and Orlando, 2001). Next, Item 6 is used as an example to further illustrate the validity of the items in the assessment tool.

***Item 6:*** *Scientists have obtained a new type of oxygen molecule (O*_4_*) by using ordinary oxygen molecules and charged oxygen ions. Which of the following statements is correct? (A) O*_4_ *is an uncharged molecule; (B) One O*_4_ *molecule contains two O*_2_ *molecules; (C) The properties of O*_4_ *are completely the same as those of O*_2_*; (D) The mixture of O*_4_ *and O*_2_ *forms a pure substance*.

After being confirmed by teachers and validated by an expert panel, Item 6 measures two attributes: A1 (Concepts) and A2 (Representation). The RMSEA value for Item 6 is 0.007 (< 0.1), the difficulty coefficient is 0.673 (within the range of 0.3–0.9), the guessing parameter is 0.1790 (< 0.5), and the slipping parameter is 0.1667 (< 0.4). After deleting this item, Cronbach's alpha of the assessment tool (0.886) did not increase. The Pearson correlation coefficient between the average score of Item 6 and the total score is 0.630 (> 0.2, *p* = 0.000). The bottom and top 27% extreme group analysis shows that the *t*-value for this item is 10.649 (> 0.3, *p* = 0.000). The above indicates that Item 6 has good content validity and construct validity.

### 2.2 Research design

**Figure 1:**
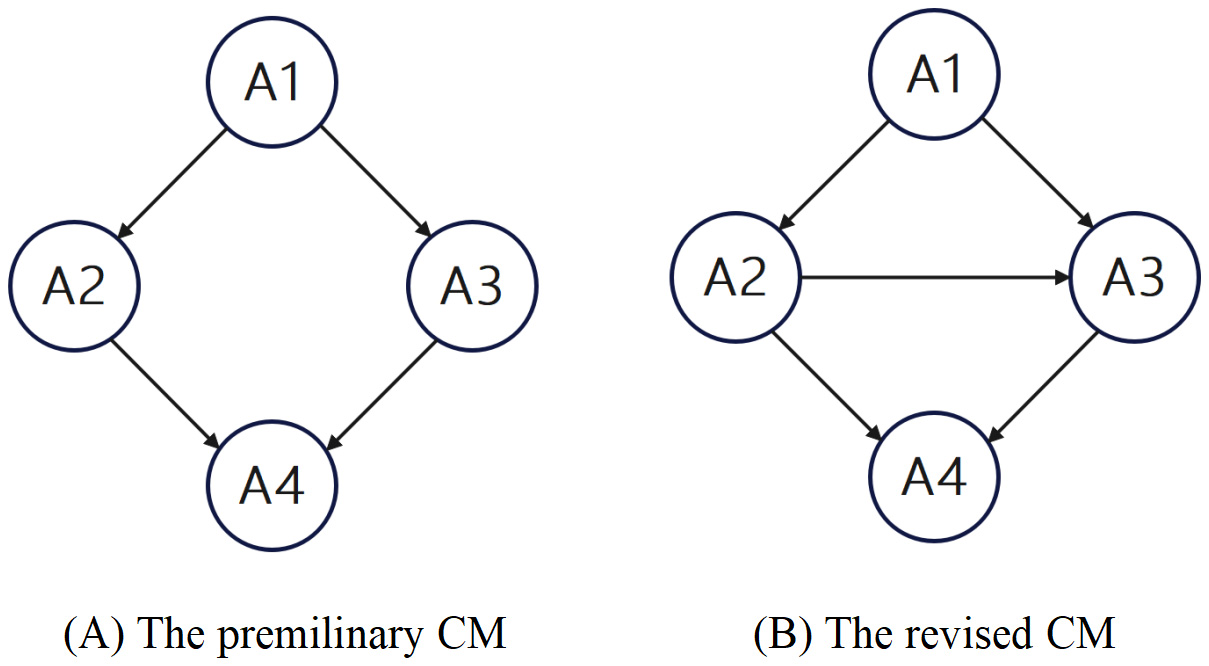
The preliminary **(A)** and the revised **(B)** CM.

We analyzed the topic “Composition and Structure of Matter” in middle school chemistry. Based on the Chemistry Curriculum Standards of Compulsory Education, multiple editions of Chinese junior high school chemistry textbooks, and chemistry items from the junior middle school academic-level test (Jilin Province, China, 2019–2024), we proposed possible cognitive attributes and their hierarchical relationships. And they were further verified by oral reports conducted with 6 students (3 top-performing group, 3 middle-performing group), using five representative chemistry items from the junior middle school academic-level test. Subsequently, a questionnaire survey was conducted to 26 chemistry teachers (mean age = 39.6 years; 63% held senior professional titles; 73% were female) from Jilin, Sichuan, and Jiangsu provinces in China. The questionnaire consists of seven items that utilize a 5-point Likert scale to evaluate the degree of teachers' agreement regarding the rationality of cognitive attributes and their hierarchical relationships. Finally, the preliminary CM for this content was developed as shown in Figure 1A. The specific identifiers, definitions, and content descriptions of cognitive attributes are presented in Table 1.

**Table 1:** Cognitive attributes.

Code	Attributes	Definitions	Content descriptions
A1	Concepts	The basic concepts of the composition and structure of matter	Understanding that matter is constituted of microscopic particles such as atoms and molecules, and is composed of macroscopic elements. Understanding the composition of atoms. Grasping fundamental concepts such as relative atomic mass, relative molecular mass, elements, atomic number, etc.
A2	Representation	Representing the composition and structure of matter	Knowing that the composition and structure of matter can be represented by symbols, such as element symbol, ionic symbol, molecular formula, and so on. Master the meaning of molecular formula, valence. Writing molecular formula. Calculating relative molecular mass, mass ratio of constituent element, and mass percentage of specific element in compound based on molecular formula.
A3	Principles	Fundamental principles of the composition and structure of matter	Understanding that material properties are related to their composition and structure. Comprehending the periodic table. Knowing the rules of electronic configuration.
A4	Application	Application of the principles of composition and structure of matter	Understanding the relationship among molecules, atoms and ions. Interpreting some related phenomena using the principles of the composition and structure of matter. Deducing composition of reactants or products based on the conservation of elements. Exploring the composition and structure of substances through methods such as experiments, imagination, reasoning, hypothesis formulation, and model building.

Using cluster sampling, we recruited 793 students from 15 intact classes across five average-level junior middle schools in Jilin, Sichuan, and Guangxi, China. The assessment tool was then used to conduct measurements. After excluding invalid responses, 752 valid cases remained, yielding a valid response rate of 94.82%. The GDINA model is employed to estimate students' mastery probabilities of cognitive attributes. In the subsequent SEM analysis, the mastery probabilities of these cognitive attributes are used as observed variables. No latent variables were included because the attribute mastery probabilities themselves served as direct indicators of students' cognitive states. The analysis focused exclusively on the path relationships among these observed variables, as specified by qualitative analysis results from teachers and experts. Thus, the model was essentially a path analysis model rather than a full latent variable SEM. This study adopts an exploratory approach because we aim to empirically refine an a preliminary CM using SEM. The following hypotheses were formulated:

H1: The preliminary CM will show poor global fit with the empirical data.

H2: Iterative model modifications guided by SEM Modification Indices (MI) will significantly improve model fit.

H3: The revised CM will achieve acceptable fit indices and yield theoretically interpretable path coefficients.

**Figure 2:**
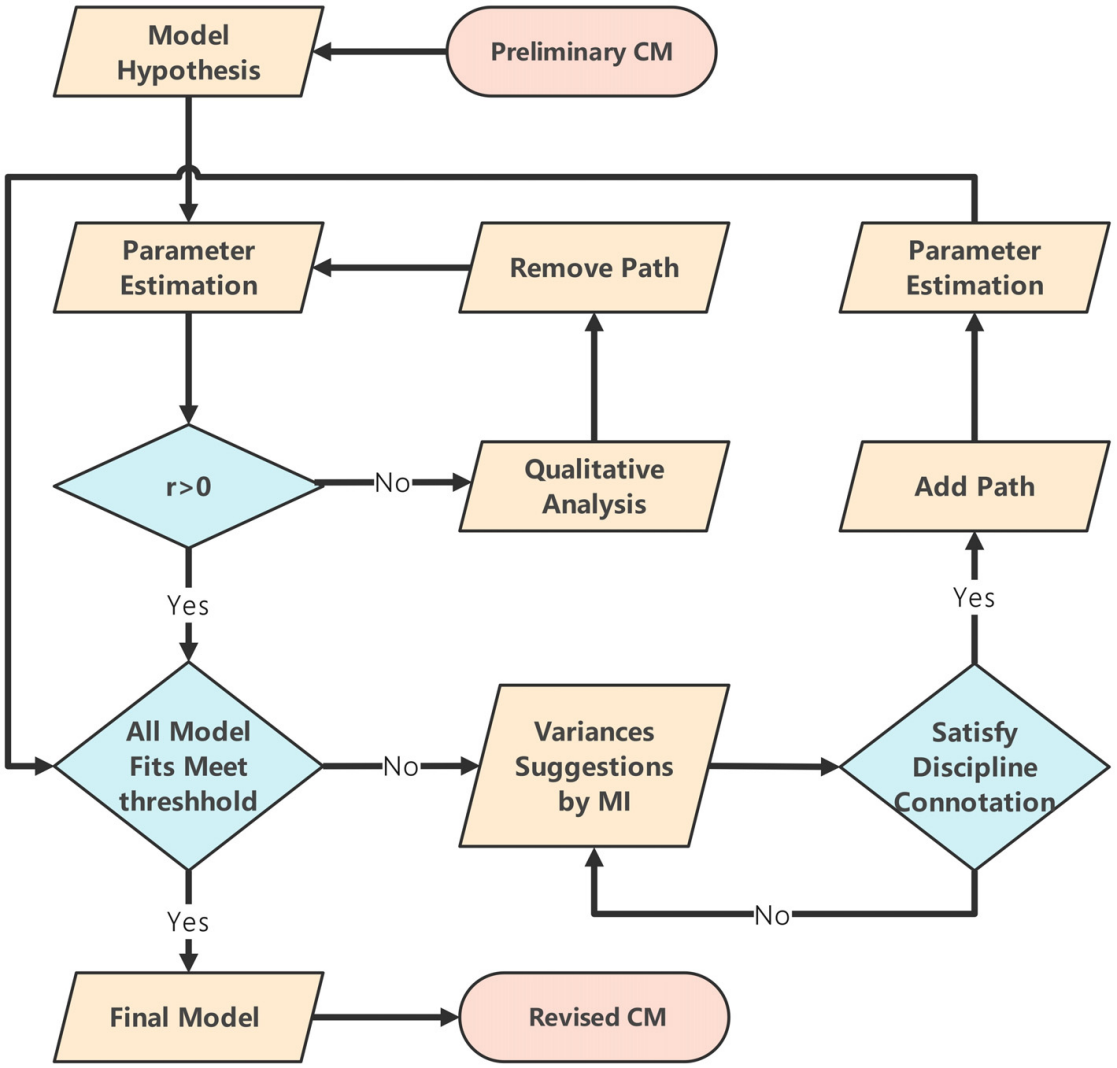
Flowchart for CM revision.

A hypothesized structural model was developed in IBM Amos 28.0 based on the preliminary CM. Students' attribute mastery probabilities were specified as observed variable, and the hierarchical interrelationships among attributes were formalized as paths relationships. Relying on quantitative parameters from SEM such as *r* and MI, as well as qualitative analysis of disciplinary connotation, a mixed-method approach is adopted for exploratory analysis. After iterative revisions, a new structural model that not only fits the data but also conforms to disciplinary connotation is obtained, and finally, a revised CM is derived. The specific strategies are as follows: (1) If *r* < 0 are detected, the associated path relationships should be removed, and parameter estimation should be performed once again. (2) Add one path at a time based on the highest MI, but only if the path makes theoretical sense. Stop adding paths when (a) the model would lose positive degrees of freedom, or (b) the added path yields a non-significant coefficient (*p* ≥ 0.05), because further changes would not improve the model. (3) The data-informed suggestions from SEM must be rigorously evaluated through theoretical scrutiny to ensure conceptual coherence and prevent overreliance on empirical patterns alone. (4) The revision process is completed when all model fit indices meet the standards and all path relationships are consistent with the underlying disciplinary connotations. The revision process is illustrated in Figure 2.

### 2.3 Results

Based on the preliminary CM, the statistical results from the expert recognition survey show that the consistency coefficient among experts was 0.81 (> 0.70), and the variance was 0.23 (< 0.25), indicating that it may still require refinement although they are reasonable (Qxley et al., 2024). Furthermore, with an HCI of 0.716 which close to the 0.70 threshold (Cui and Leighton, 2009), the attribute hierarchy appears generally reasonable yet possibly in need of refinement.

A hypothetical model was established based on the preliminary CM, with the mastery probabilities treated as observed variables and the hierarchical relationships among attributes specified as path relationships. The regression coefficients for the paths A1 → A2, A1 → A3, A3 → A4, and A2 → A4 were 0.685, 0.702, 0.594, and 0.115, respectively. All regression coefficients between cognitive attributes were statistically significant (*p* < 0.001), indicating that the hierarchical relationships were statistically rational. As shown in Supplementary Table S1, most goodness-of-fit statistics met the recommended thresholds. However, certain parameter values—including the RMSEA among absolute fit indices, RFI and TLI among incremental fit indices, PGFI, PNFI, PCFI, and χ^2^/df among parsimonious fit indices—fell outside the acceptable range. Obviously, not all goodness-of-fit statistics met the recommended thresholds, suggesting discrepancies between the CM implied by the actual response data and the preliminary CM. This indicates the necessity of revising the preliminary CM.

The MI values obtained from the first-round parameter estimation of the hypothetical model are presented in Table 2. The “Suggestion” column lists proposed additions of covariance or path relationships within the model, while the MI values represent the expected reduction in the chi-square statistic resulting from these modifications. A higher MI value indicates a greater potential improvement in model fit following the corresponding modification suggestions (Saris et al., 1987; Bagozzi and Yi, 1988). The MI value for e2↔e3 is the highest (13.133), indicating that establishing a covariance relationship between e2 and e3 would yield the most substantial improvement in the overall fit of the hypothetical model. The MI values for A2 → A3 (12.296) and A3 → A2 (12.008) suggest that introducing a path relationship in either direction would also substantially improve the model fit. From the perspective of chemistry disciplinary connotation, A2 (Representation) pertains to the ability to express and discern the composition and structure of substances, and corresponds to the concrete operational level of chemistry-related skills. In contrast, A3 (Principle) involves fundamental theories regarding composition and structure, aligning with the abstract cognitive level in chemistry. From the perspective of cognitive development laws of progressing from the superficial to the profound, A3 should be a more advanced level than A2. Therefore, the path A2 → A3 should be established.

**Table 2:** The modification indices for the preliminary CM.

Suggestion	MI	Par change
e2↔e3	13.113	0.008
e4↔e1	4.723	0.002
A3←A2	12.296	0.108
A2←A3	12.008	0.138
A4←A1	4.732	0.237

The preliminary CM encompasses four attributes, resulting in the number of observable variables being 4. By applying the formula *k*(*k* + 1)/2, where *k* represents the number of observed variables, it can be calculated that the number of data points is 10. The number of parameters to be estimated in the hypothetical model is eight, including four regression weights for the paths and four variances associated with the attributes. Therefore, if two modification suggestions from the MI were adopted simultaneously, the number of parameters to be estimated would increase to 10 (8 original parameters plus 2 additional ones). Under this condition, the degrees of freedom (df) would be 0, calculated as the difference between the number of data points and the number of parameters to be estimated (df = 10 - 10 = 0). This outcome does not satisfy the *t*-criterion requirement (df > 0), rendering the model unidentifiable and thus inestimable. Therefore, only one model modification suggestion can be implemented at a time in this study.

Following the principle of prioritizing modifications with higher MI values, the covariance relationship between e2 and e3 was first established. After incorporating the suggested modification, all overall goodness-of-fit statistics of the model met the recommended thresholds. The estimated covariance between e2 and e3 was 0.008 (> 0), reaching statistical significance at the 0.001 level. This suggests a strong association between A2 and A3, although the directionality of the influence remains undetermined. Subsequently, the covariance relationship between e2 and e3 was removed, and a directional path from A2 to A3 was established. After the establishment of the A2 → A3 path, all parameter estimation results remained entirely consistent with those obtained under the e2↔e3 covariance relationship. Therefore, the establishment of the A2 → A3 path was deemed appropriate. SEM analysis of the revised CM indicated that all overall goodness-of-fit indices satisfied the recommended thresholds, and no further modification suggestions were identified based on the MI values. The estimated path coefficients and their significance levels supported the validity of the hierarchical relationships among the cognitive attributes. A classic cognitive diagnostic analysis conducted using the GDINA model indicates that the HCI value has significantly improved, rising from 0.716 in the preliminary CM to 0.921 in the revised CM. The revised CM is illustrated in Figure 1B.

## 3 Discussion

### 3.1 Psychological analysis

Bruner's theory of cognitive representation (Bruner, 1966) describes a progression from enactive to iconic to symbolic modes. In chemistry learning, this translates to moving from concrete observations to iconic/symbolic representations (e.g., chemical formulas) and then to abstract principles (e.g., conservation of mass). In this paper, the mixed-method based on SEM identified a direct A2 (representation) → A3 (principle) path that was absent in the preliminary CM. This finding aligns with Bruner's iconic-to-abstract sequence: students first develop representational skills (A2), which then serve as a cognitive foundation for understanding theoretical principles (A3). Similar representational-to-principle hierarchies have been reported in chemistry education research (e.g., Chandrasegaran et al., 2007). Theoretically, when constructing a CM for a chemistry topic, the hierarchy should explicitly reflect that representational competence is a prerequisite for principle-based reasoning. This insight informs both the development of CMs in chemistry education and the optimization of chemistry teaching practices.

### 3.2 Advantages

#### 3.2.1 Comparison with classical methods

Classical methods for CM specification rely heavily on expert judgment. This process may be influenced by cognitive biases such as the primacy effect (Rubínová et al., 2022; Rubínová and Price, 2024). This method integrates quantitative and qualitative methodologies, constituting a mixed-methods approach that not only upholds the cognitive-psychological validity of the CM but also mitigates specification error arising from researchers' cognitive biases. The validation criteria in classical CD have relatively broad acceptable ranges (e.g., HCI > 0.7, *R*^2^ > 0.6), meaning a CM with some unreasonable elements could still pass validation. Moreover, it fails to provide a specific revision plan. In contrast, the SEM-based approach supplies multiple fit indices (absolute, incremental, and parsimonious) and MI that pinpoint specific misspecified paths. It can also yield path coefficients and significance tests for relationships among attributes, allowing researchers to evaluate the strength and direction of hypothesized causal effects. Furthermore, SEM can be implemented through graphical software (e.g., IBM Amos), making it accessible without advanced programming skills.

#### 3.2.2 Empirical support

We have not identified direct empirical studies on using SEM to revise the CM. However, our previous work (Lin et al., 2025) successfully used SEM to revise the Q-matrix in middle school chemistry, showing that SEM can be applied to CD, and it is capable of identifying the relationships among various factors within a structure. The present study extends this approach to the CM—examining the inter-factor hierarchy of cognitive attributes—which is qualitatively consistent with our prior research. Moreover, our current results provide empirical support: after revision by the mixed-method based on SEM, the goodness-of-fit between the data and the model has been improved. Thus, this study offers initial real-data evidence that SEM can be employed to revise the CM.

### 3.3 Limitations and future directions

#### 3.3.1 Measurement error

This study employed a two-stage analytical approach. In the first stage, mastery probabilities were estimated using the GDINA model. These estimates carry standard errors but were treated as error-free observed variables in the second-stage SEM. This may lead to underestimated standard errors of path coefficients and overly confident inferences about attribute relationships (Skrondal and Laake, 2001). Future studies should adopt a full latent variable SEM approach that simultaneously estimates both the measurement model and the structural model, thereby properly accounting for measurement error. Alternatively, a bias-correction method for two-stage estimates could be explored.

#### 3.3.2 Sample generalizability

The sample was drawn from five average-level junior middle schools in three regions of China (Jilin, Sichuan, Guangxi). Although cluster sampling was used, the sample may not represent all school types (e.g., rural vs. urban, high-performing vs. low-performing). To establish that the revised CM reflects a stable cognitive structure rather than sample-specific artifacts, future research should conduct cross-validation with independent samples.

## 4 Practical implications

In our study, the direct A2 → A3 relationship suggested by SEM revealed a cognitive sequence—representation before principle—that was initially overlooked. This illustrates how the method can uncover latent cognitive structures that qualitative analysis alone might miss, thereby informing more effective instructional sequencing. The improved precision of the CM enhances the diagnostic utility of cognitive assessments and teachers can confidently structure learning progressions. Furthermore, a better-specified attribute hierarchy leads to more reliable classification of students' mastery profiles. In turn, this allows educators to design targeted interventions: for instance, identifying whether a student's difficulty in solving application problems stems from inadequate representational skills or insufficient principle knowledge. The method thus contributes to the development of more valid diagnostic assessments that can directly inform differentiated instruction.

Beyond its specific application in middle school chemistry education, as outlined above, the SEM methodology adopted in this study offers broader methodological implications and practical value. Although this study is grounded in secondary school chemistry curricula, the methodological framework developed herein demonstrates broad applicability across science disciplines (e.g., physics, biology) or even non-STEM subjects, enhancing the rigor and validity of CM across disciplines. As such, it holds significant practical implications for advancing cross-disciplinary curriculum reform, particularly in supporting individualized instruction and targeted remediation. Consequently, the SEM-based revision method presented here offers a powerful tool for advancing CD research, with the potential to improve both the scientific foundation of CM and its practical application in educational settings.

## Data Availability

The datasets presented in this study can be found in online repositories. The names of the repository/repositories and accession number(s) can be found in the article/supplementary material.
