# Supplementary material for: Revise the cognitive model using structural equation modeling
Source: Front Psychol. 2026 May 18;17:1758968. doi: 10.3389/fpsyg.2026.1758968 (PMC13223082; doi:10.3389/fpsyg.2026.1758968)
Supplement: Supplementary file 1 [file Data_Sheet_1.pdf]

Supplementary Material

TABLE S1 | The overall goodness-of-fit statistics of the premilinary CM.

Statistical measures and their critical values	Estimated results	Meets threshold or not
Absolute fit indices		
RMR（< 0.05）	0.003	Yes
RMSEA（< 0.08）	0.105	No
GFI（> 0.90）	0.988	Yes
AGFI（> 0.90）	0.939	Yes
Incremental fit indices		
NFI（> 0.90）	0.941	Yes
RFI（> 0.90）	0.823	No
IFI（> 0.90）	0.947	Yes
TLI（> 0.90）	0.839	No
CFI（> 0.90）	0.946	Yes
Parsimonious fit indices		
PGFI（> 0.50）	0.198	No
PNFI（> 0.50）	0.314	No
PCFI（> 0.50）	0.315	No
CN（> 200）	242	Yes
χ^2/df（< 2.00）	55.666	No
